# Perioperative factors influencing the difficulty of retroperitoneal laparoscopic adrenalectomy: a single-center retrospective study

**DOI:** 10.1186/s12894-022-00976-y

**Published:** 2022-02-17

**Authors:** Jinyao Wang, Bin Yang, Shiwei Sun, Yangang Zhang

**Affiliations:** 1 Shanxi Bethune Hospital, Shanxi Academy of Medical Sciences, Tongji Shanxi Hospital, Third Hospital of Shanxi Medical University, Taiyuan, 030032 China; 2 Tongji Hospital, Tongji Medical College, Huazhong University of Science and Technology, Wuhan, 430030 China

**Keywords:** Laparoscopic adrenalectomy, Surgical difficulty, Perioperative factors

## Abstract

**Purpose:**

Identifying patients in whom adrenalectomy may be more difficult can help with surgical decision-making. This study investigated the perioperative factors affecting the difficulty of retroperitoneal laparoscopic adrenalectomy (RLA).

**Methods:**

Sixty-eight patients who underwent RLA at our hospital between December 1, 2020 and May 1, 2021 were included. The difficulty of RLA was assessed by operating time and intraoperative blood loss. We analyzed the relationship between surgical difficulty and patient sex, age, and body mass index, pathological type, tumor side, tumor size, distance from the lower pole of the adrenal tumor to the upper pole of the kidney (DAK), and distance from the lower pole of the adrenal tumor to the renal pedicle (DARP).

**Results:**

Mean operating time was 105.38 ± 33.31 min and mean intraoperative blood loss was 32.28 ± 22.88 ml. Univariate linear regression analysis showed that age (*P* = 0.047), tumor size (*P* = 0.002), DAK (*P* = 0.002), and DARP (*P* < 0.001) were significantly correlated with a longer operating time. Univariate logistic regression analysis showed that DARP (*P* = 0.001), DAK (*P* = 0.001), tumor size (*P* = 0.002), and age (*P* = 0.033) were significantly correlated with a longer operating time. Multivariate logistic regression indicated that DARP (OR 5.341; 95% CI 1.704–16.739; *P* = 0.004), and tumor size (OR 4.433; 95% CI 1.434–13.709; *P* = 0.010) were independent predictors of operating time.

**Conclusion:**

Age, tumor size, DAK, and DARP were predictors of the difficulty of RLA. Older age, lower DARP and DAK, and a larger tumor size were associated with a longer operating time. DARP and tumor size were independent predictors of surgical difficulty.

## Introduction

Compared with traditional open surgery, laparoscopic adrenalectomy has become the gold standard for the surgical treatment of adrenal disease because of its good exposure and precise local anatomy [1]. At the same time, compared with the transperitoneal approach, the retroperitoneal approach can avoid intraoperative intestinal interference and postoperative intestinal obstruction as well as other related complications, so is conducive to recovery after surgery. However, the retroperitoneal approach has a relatively small operating space with unclear anatomical landmarks and is technically difficult to perform. Moreover, retroperitoneal adrenalectomy relies more heavily on the accuracy of the surgical approach, and the incidence of complications is estimated to be 3%–20% [2, 3]. Therefore, evaluation of the difficulty of surgery preoperatively is important in terms of avoiding iatrogenic harm, reducing intraoperative blood loss, shortening the operating time, lowering the risk of postoperative complications, and minimizing the length of hospital stay. In this study, we investigated patients who have undergone retroperitoneal adrenalectomy for adrenal tumors at our institution in recent years with the aim of identifying factors that affect the difficulty of this operation.

## Materials and methods

### Study population and selection process

All patients were consecutive cases. Of these, 18 patients were excluded because they did not meet the inclusion criteria, 11 patients were excluded because the preoperative CT or MRI study was not in the radiology system archives, and 2 patients were also excluded during follow-up due to incomplete data or lost follow-up.

Inclusion criteria: (1) Preoperative CT suggested benign adrenal tumor; (2) A series of routine laboratory tests were performed preoperatively to determine the hormone activity of the tumor; (3) Patients who were treated by retrolaparoscopic surgery and by the same senior surgeon.

Exclusion criteria: (1) Patients with a history of lumbar surgery; (2) Patients who cannot undergo retrolaparoscopic surgery; (3) Patients who were confirmed malignant mass by postoperative pathology; (4) Patients with maximum tumor diameter > 60 mm; (5) Patients who underwent multiple operations simultaneously.

All sixty-eight patients who underwent retroperitoneal laparoscopic adrenalectomy between December 1, 2020 and May 1, 2021 at our institution were enrolled. The data were collected retrospectively. The patients underwent surgery performed by a specialist team at Shanxi Bethune Hospital, which specializes in the treatment of adrenal tumors. This surgical team has performed more than 100 laparoscopic adrenalectomies. All patients underwent preoperative computed tomography (CT) and/or magnetic resonance imaging (MRI) and a series of routine laboratory investigations to determine the hormonal activity of the tumor.

We anticipated that operating time and intraoperative blood loss would be the main determinants of the degree of difficulty level of LTA. We analyzed the relationship between surgical difficulty and patient sex, age, and body mass index (BMI), pathological type of tumor, tumor side (left/right), tumor size, distance from the lower pole of the adrenal tumor to the upper pole of the kidney (DAK), and distance from the lower pole of the adrenal tumor to the renal pedicle (DARP). Tumor size, DAK, and DARP were estimated on the CT/MRI scans obtained before surgery. Tumor size was indicated by the maximum tumor diameter.

This study was approved by the Ethical Review Board of Shanxi Medical University. All procedures followed the ethical standards of the responsible committee on human experiments (institutional and national) and were performed in accordance with the Declaration of Helsinki. Written informed consent was obtained from all patients before surgery.

### Statistical analysis

All continuous variables are shown as the mean and standard deviation and were tested for normality using the chi-squared test. Categorical variables were compared using the chi-squared test. The effects of various perioperative factors on operating time and intraoperative blood loss were analyzed by single factor linear regression and single factor logistic analysis. Multivariate linear regression analyses were performed to identify factors related to procedural difficulty. One-way analysis of variance was used to compare the means of multi-group measurement data. The Student–Newman–Keuls method was used for pairwise comparison of means between groups. Correlation analysis of two variables was performed using the Spearman method. All statistical analyses were performed using SPSS software version 26.0 (IBM Corp., Armonk, NY). A *P* value < 0.05 was considered statistically significant.

## Results

Sixty-eight patients (29 men, 39 women) with benign adrenal tumors (45 left-sided, 23 right-sided) were enrolled in the study. The mean patient age was 51.18 ± 11.62 years (range 22–70). The mean tumor size was 27.03 ± 11.95 mm (range 10.00–60.00). Mean BMI (kg/m^2^) was 24.83 ± 3.22 (range 16.13–32.20). The mean DAK was 14.31 ± 12.58 mm (range − 52.00 to 14.00) and the mean DARP was 29.25 ± 10.79 mm (range − 4.00 to 64.00). The mean operating time was 105.38 ± 33.31 min (range 41–184) and the mean estimated intraoperative blood loss was 32.28 ± 22.88 ml (range 15–150) (Table 1).

**Table 1:** Clinical characteristics of the studied group

Variable	Total cohort	Operative time		*P* value
	(n = 68)	≤ 120 min (n = 34)	> 120 min (n = 34)	
Age (year)	51.18 (± 11.621)	48.27 ± 11.371	54.09 ± 11.288	0.04
Sex				0.09
Male	29 (42.6)	11 (37.9)	18 (62.1)	
Female	39 (57.4)	23 (59.0)	16 (41.0)	
BMI (kg/m^2^)	24.828 (± 3.219)	24.632 ± 3.3	25.025 ± 3.173	0.6
Side				0.4
Left	45 (66.2)	24 (53.3)	21 (46.7)	
Right	23 (33.8)	10 (43.5)	13 (56.5)	
Pathology				0.5
NFAT	36 (52.9)	19 (52.8)	17 (47.2)	
PA	11 (16.2)	6 (54.5)	5 (45.5)	
CS	13 (19.1)	7 (53.8)	6 (46.2)	
Others	8 (11.8)	2 (25.0)	6 (75.0)	
TD (mm)	27.03 (± 11.946)	22.00 ± 7.808	32.06 ± 13.291	< 0.001
DAK (mm)	− 14.31 (± 12.577)	− 9.77 ± 12.497	− 18.86 ± 11.065	0.002
DARP (mm)	29.25 (± 10.789)	34.00 ± 9.165	24.50 ± 10.288	< 0.001

Data are presented as mean (± standard deviation) for continuous variables and as n (%) for categorical variables. Comparisons between the length of operation groups were made using the independent samples t-test for continuous variables and the χ^2^ test for categorical variables

*BMI* body mass index, *NFAT* nonfunctioning adrenal tumor, *PA* primary aldosteronism, *CS* Cushing’s syndrome; Others include myelolipoma, cyst, ganglioneuroma, lymphangioma, teratoma, *TD* tumor diameter, *DAK* distance from lower pole of adrenal tumor to upper pole of kidney, *DARP* distance from adrenal tumor to renal pedicle

**Fig. 1:**
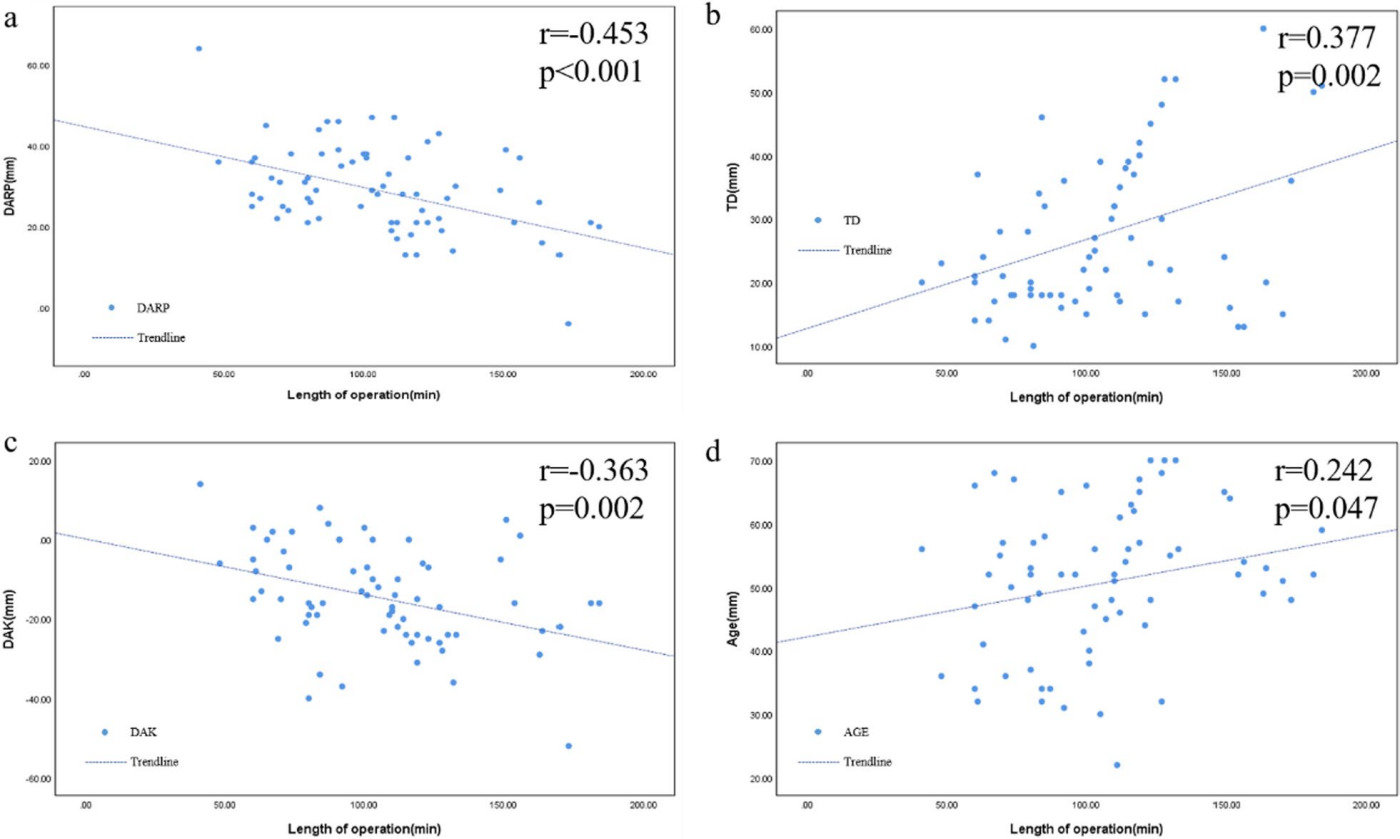
Scatter Plots of risk factors and the length of operation (**a** DARP. **b** TD. **c** DAK. **d** age). (*TD* tumor diameter, *DAK* distance from lower pole of adrenal tumor to upper pole of kidney, *DARP* distance from adrenal tumor to renal pedicle)

**Fig. 2:**
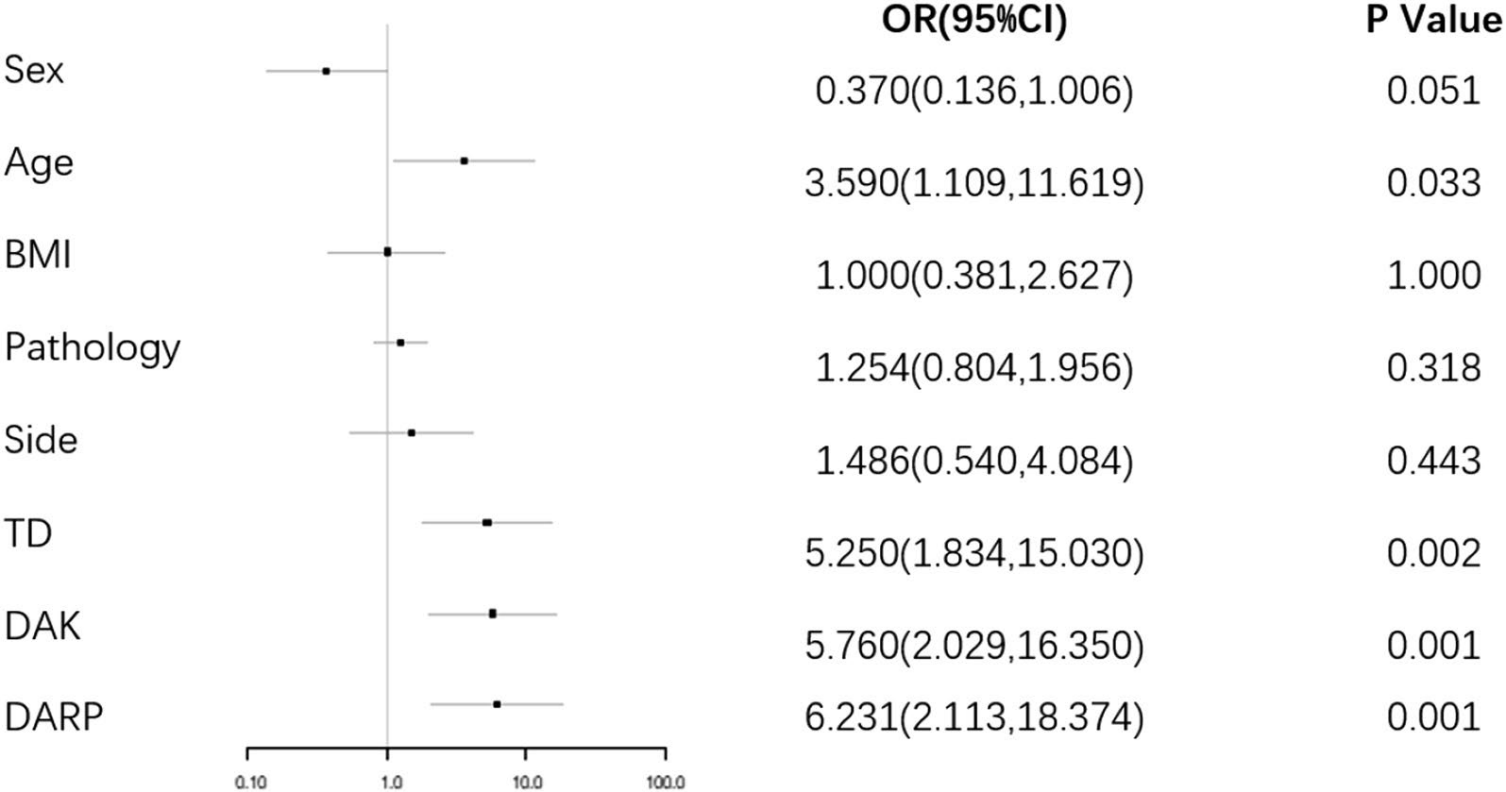
Forest plot of risk factors and the length of operation, *TD* tumor diameter, *DAK* distance from lower pole of adrenal tumor to upper pole of kidney, *DARP* distance from adrenal tumor to renal pedicle

Many perioperative factors had varying degrees of influence on operating time (Table 2). Univariate linear regression analysis showed that age (r = 0.242, *P* = 0.047), tumor size (r = 0.377, *P* = 0.002), DAK (r = − 0.363, *P* = 0.002) and DARP (r = − 0.453, *P* < 0.001) were significantly correlated with operating time (Fig. 1). Univariate logistic regression analysis showed that DARP (odds ratio [OR] 6.231, 95% confidence interval [CI] 2.113–18.374; *P* = 0.001), DAK (OR 5.76, 95% CI 2.029–16.35; *P* = 0.001), tumor size (OR 5.25, 95% CI 1.834–15.03, *P* = 0.002), and age (OR 3.59, 95% CI 1.109–11.619; *P* = 0.033) were significantly correlated with operating time (Fig. 2). Multivariate logistic regression showed that DARP (OR 5.341, 95% CI 1.704–16.739; *P* = 0.004) and tumor size (OR 4.433, 95% CI 1.434–13.709; *P* = 0.010) were independent predictors of operating time (Table 2). However, there was no significant interaction between any of these factors and intraoperative blood loss (Table 3).None of the patients had serious complications such as conversion and massive bleeding. No patients had blood transfusion due to bleeding, and all patients were hospitalized for 3–21 days, with an average of 8.09 days. Fourteen patients were hospitalized for more than 9 days (P75). There were 5 types of complications in 10 patients. Among them, systemic inflammatory response syndrome in 1 case, postoperative hypokalemia in 4 cases, incomplete intestinal obstruction in 1 case, lower limb venous thrombosis in 2 cases, pressure ulcers in 2 cases. According to the modified Clavien classification, 7 cases were grade I, 2 case were Grade IIIA and 1 case was grade IVB. (Table 4).

**Table 2:** Associations of clinical characteristics on operative time

	Univariate			Multivariate		
	OR	(95% CI)	*P* value	OR	(95% CI)	*P* value
Age (year)	1.047	1.002–1.095	0.04	1.049	0.988–1.114	0.1
Sex						
Male	1		–	1		–
Female	0.370	0.136–1.006	0.051	0.312	0.085–1.150	0.08
BMI (kg/m^2^)	1.039	0.895–1.207	0.6	0.955	0.763–1.195	0.7
Side						
Left	1		–	1		–
Right	1.486	0.510–4.084	0.3	0.924	0.235–3.638	0.9
Pathology						
NFAT	1		–	1		–
PA	0.931	0.240–3.612	0.9	0.963	0.173–5.348	0.9
CS	0.979	0.518–1.849	0.9	0.561	0.128–2.452	0.4
Others	1.497	0.841–2.663	0.2	1.257	0.639–2.470	0.5
TD (mm)	1.090	1.034–1.149	0.001	1.064	1.005–1.126	0.03
DAK (mm)	0.935	0.892–0.980	0.005	0.993	0.910–1.083	0.9
DARP (mm)	0.896	0.840–0.956	0.001	0.919	0.859–0.984	0.02

**Table3:** Associations of clinical characteristics on amount of bleeding

	Univariate regression analysis				
	B	SE	r	t	*P* value
Sex	− 1.589	5.677	− 0.034	− 0.28	0.8
Age	0.211	0.241	0.107	0.877	0.4
BMI	0.048	0.875	0.007	0.055	1.0
Pathology	− 0.424	2.572	− 0.020	− 0.165	0.9
Side	− 0.159	5.909	− 0.003	− 0.027	1.0
TD	0.431	0.230	0.225	1.876	0.06
DAK	− 0.431	0.218	− 0.237	− 1.983	0.052
DARP	− 0.335	0.258	− 0.158	− 1.300	0.2
Length of operation	0.053	0.084	0.078	0.634	0.5

**Table 4:** Classification of complications

	No (%)
Systemic inflammatory response syndrome	1 (1.5)
Postoperative hypokalemia	4 (5.9)
Incomplete intestinal obstruction	1 (1.5)
Deep venous thrombosis	2 (2.9)
Pressure sore	2 (2.9)

## Discussion

The first case of laparoscopic adrenalectomy was reported by Gagner et al. in 1992 [4]. Since then, retroperitoneal laparoscopic adrenalectomy has become a widely used surgical method. Several scoring systems, such as the RENAL score and Padua score [5], can be used by renal surgeons to optimize preoperative planning and surgical technique and to provide guidance for improving the prognosis [6]. However, at present, there is no scoring system that can be used to predict the difficulty of surgery for adrenal tumors. Creation of such a scoring system would help to guide the selection of surgical methods and predicting the difficulty of surgery. In 2021, Alberici et al. devised a scoring method that could be used to predict the difficulty of transabdominal laparoscopic adrenalectomy using three models and found that this score could also predict a complicated postoperative course [7]. Furthermore, Vrielinkom et al. reported that retroperitoneal laparoscopic adrenalectomy could reduce the operating time, amount of blood loss, and complication rate in comparison with transabdominal laparoscopic adrenalectomy [8]. Therefore, we sought to identify the perioperative factors affecting the difficulty of retroperitoneal laparoscopic adrenalectomy.

The adrenal gland is located in the upper pole of the kidney. CT is the most common imaging method used for diagnosis of adrenal tumors [9] and provides appropriate anatomical landmarks that can be used as a reference for precise localization. In this study, the renal pedicle and the plane of the upper renal pole were selected as the positioning markers for measurement of DAK and DARP for two reasons. First, based on the relative clarity and fixation of these anatomical markers on the CT image, it was easy to obtain the relative position of the adrenal gland. Second, based on our clinical experience, we consider that surgery for relatively low adrenal tumors is more difficult than that for high adrenal tumors. In this study, we found that DAK and DARP were the strongest predictors of the difficulty of retroperitoneal laparoscopic adrenalectomy, specifically, the smaller the DARP and DAK, the longer the operating time.

We also examined the correlations of other indices with operating time and intraoperative blood loss and confirmed a strong correlation of operating time with tumor size but not with BMI. In 2010, Erbil et al. investigated the effect of retroperitoneal fat mass on the prognosis in patients undergoing laparoscopic adrenalectomy [10]. As in our study, they did not find a significant correlation between BMI and operating time (*P* = 0.51). However, they found a significant correlation between operating time and the ratio of retroperitoneal fat area to adrenal mass area (*P* = 0.0001). In their patients with high BMI, the operating time was longer and the incidence of complications was higher in those with a high retroperitoneal fat ratio than in those with a low retroperitoneal fat ratio. Therefore, retroperitoneal fat is considered to be a more useful parameter than BMI in predicting the outcome of laparoscopic adrenalectomy. A study by Rao et al. in 2016 explored the role of tumor size in laparoscopic surgery for adrenal pheochromocytoma [11]. The operating time, intraoperative blood loss, and complications of larger tumors were similar to those of smaller tumors, and they concluded that tumor size did not affect the outcome of laparoscopic adrenalectomy for pheochromocytoma. Natkaniec et al. investigated 275 patients who underwent retroperitoneal laparoscopic adrenalectomy and identified that tumor size as well as age, sex, and pathological tissue type were predictors of surgical difficulty [12]. They also found that the laterality of the tumor, BMI, American Society of Anesthesiologists' grade, and previous abdominal surgery had no effect on the short-term results. Lindeman et al. determined that transabdominal adrenalectomy using image localization markers was a better treatment [13]. The distance from the skin to Gerota’s fascia (S-GF), the distance between the upper border of the adrenal gland and kidney, the distance between the adrenal gland and the 12th rib were measured respectively, 12 costal and perirenal fat distance, 12 iliac spine and perirenal fat distance (PNF). The associations of these characteristics, as well as BMI, sex, age, tumor size, and diagnosis, with operating time and estimated blood loss were examined using Pearson’s correlation or analysis of variance. Patients with a high BMI were more likely to undergo transabdominal adrenalectomy and there was a significant association of larger tumor size with longer operating time (r = 0.341). In bivariate analysis, S-GF and PNF were moderately correlated with operating time (r = 0.464 and r = 0.494, respectively). The posterior adiposity index (PAI) was obtained by adding S-GF and PNF and found to be closely related to the operating time (r = 0.590) but not to the estimated amount of bleeding. Larger lesions (*P* = 0.025) and an increased PAI (*P* = 0.019) predicted a longer operating time.

The type of surgical scope chosen for retroperitoneal adrenalectomy depends on the difficulty of the operation. Some authors have proposed different surgical methods for small adrenal tumors with partial dissociation and those with full dissociation of the adrenal glands [14]. No matter what method is used, the main considerations are accuracy, comprehensiveness, avoid omission, and the shortest operating time possible to achieve the goals of minimally invasive treatment and rapid rehabilitation.

## Conclusions

In this study, patient age, tumor size, DAK, and DARP were the strongest predictors of the difficulty of retroperitoneal laparoscopic adrenalectomy. Older age, shorter DARP and DAK, and a larger tumor size were associated with a longer operating time. DARP and tumor size were also independent predictors of the difficulty of retroperitoneal laparoscopic adrenalectomy. Distance between adrenal tumor and anatomical mark based on the anatomical position on CT is a feasible method for evaluation of the difficulty of surgery and selection of the best surgical approach.

## Data Availability

The datasets used and/or analyzed during the current study are available from the corresponding author on reasonable request.
